# A Perspective on Missing Aspects in Ongoing Purification Research towards *Melissa officinalis*

**DOI:** 10.3390/foods12091916

**Published:** 2023-05-08

**Authors:** Roberto Castro-Muñoz, Grzegorz Boczkaj, René Cabezas

**Affiliations:** 1 Tecnologico de Monterrey, Campus Toluca, Avenida Eduardo Monroy Cárdenas 2000 San Antonio Buenavista, Toluca de Lerdo 50110, Mexico; 2 Department of Sanitary Engineering, Faculty of Civil and Environmental Engineering, Gdansk University of Technology, 11/12 Narutowicza St., 80-233 Gdansk, Poland; 3 Departamento de Química Ambiental, Facultad de Ciencias, Universidad Católica de la Santísima Concepción, Concepción 4090541, Chile

**Keywords:** green chemistry, phytochemicals, bioactive substances, chemical profiling, antioxidants

## Abstract

*Melissa officinalis* L. is a medicinal plant used worldwide for ethno-medical purposes. Today, it is grown everywhere; while it is known to originate from Southern Europe, it is now found around the world, from North America to New Zealand. The biological properties of this medicinal plant are mainly related to its high content of phytochemical (bioactive) compounds, such as flavonoids, polyphenolic compounds, aldehydes, glycosides and terpenes, among many other groups of substances. Among the main biological activities associated with this plant are antimicrobial activity (against fungi and bacteria), and antispasmodic, antioxidant and insomnia properties. Today, this plant is still used by society (as a natural medicine) to alleviate many other illnesses and symptoms. Therefore, in this perspective, we provide an update on the phytochemical profiling analysis of this plant, as well as the relationships of specific biological and pharmacological effects of specific phytochemicals. Currently, among the organic solvents, ethanol reveals the highest effectiveness for the solvent extraction of precious components (mainly rosmarinic acid). Additionally, our attention is devoted to current developments in the extraction and fractionation of the phytochemicals of *M. officinalis*, highlighting the ongoing progress of the main strategies that the research community has employed. Finally, after analyzing the literature, we suggest potential perspectives in the field of sustainable extraction and purification of the phytochemical present in the plant. For instance, some research gaps concern the application of cavitation-assisted extraction processes, which can effectively enhance mass transfer while reducing the particle size of the extracted material *in situ*. Meanwhile, membrane-assisted processes could be useful in the fractionation and purification of obtained extracts. On the other hand, further studies should include the application of ionic liquids and deep eutectic solvents (DES), including DESs of natural origin (NADES) and hydrophobic DESs (hDES), as extraction or fractionating solvents, along with new possibilities for effective extraction related to DESs formed *in situ*, assisted by mechanical mixing (mechanochemistry-based approach).

## 1. Introduction

**Figure 1:**
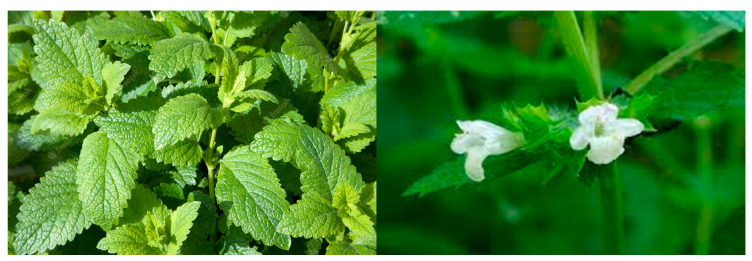
Digital images of the physical aspects of a typical *M. officinalis* plant. Note: photos taken by the authors.

Since ancient times, many plants belonging to different families have been used for their medicinal properties to alleviate specific symptoms and illnesses in human beings. It is known that over 80% of the global population still utilizes plants and herbs to treat diseases as part of traditional medicine [1]. As illustrated in Figure 1, *Melissa officinalis* L. presents wrinkled, ovate, medium green leaves (up to 3 inches long), which grow in pairs on square stems rising to 2 inches tall. Over the summer, tiny, two-lipped, white flowers appear on the leaf axils. Depending on their type, the plants mostly contain active phytochemicals, including alkaloids, flavonoids, glycosides, phenolic compounds, polysaccharides, saponins, tannins, proteins, volatile oils, gingerols and capsaicins, among many other specific substances (e.g., minerals and vitamins) that are needed for some specific metabolic pathways in humans [2,3,4,5,6,7,8].

*M. officinalis* is a typical plant that has been used for ethno-medical and therapeutical aims, including for antibacterial, antioxidant, antidiabetic, anti-inflammatory, antispasmodic, anti-insomnia and even antidepressive purposes [9]. For instance, some countries, such as Austria, Brazil, Denmark, Croatia and Iran, have utilized several parts of the plant to alleviate gastrointestinal issues, migraine, rheumatism and depression, among other illnesses. Table 1 gives complete information about the ethnopharmacological applications of *M. officinalis* in different countries. Although *M. officinalis* originated primarily in Southern Europe, it is now found around the world, from North America to New Zealand [9]. This plant is reported to contain different phytochemical substances, such as volatile and aromatic bioactives, triterpenes, flavonoids, phenolic compounds and acids, to which such therapeutic effects have been credited. In this plant, the active phytochemicals can be found in wide varieties among its different parts, including the roots, seeds, leaves, skin, flowers and the entire plant [10]. 

However, in addition to its therapeutic purposes, *M. officinalis* has also been used for culinary purposes due to its abundance of aromatic and volatile substances, such as geranial, neral, citronellal and geraniol, to mention just a few of them. The plant and its extracts have been involved in flavoring, garnishing, drink and beverage preparation, herbal oil fabrication, soups, meat dishes and sauces [12].

Nowadays, great efforts have been made to determine the phytochemical composition and substance profiling related to this medicinal plant. This is also supported by the current trend of finding specific metabolites and phytochemicals that are challenging to chemically synthesize. The interest in medicinal plants relies on their primary role as a source of biologically active substances and their usage, after their successful recovery and purification, in products such as supplements, pharmaceuticals and nutraceuticals, as stated by experts in the field [13,14,15]. In this perspective, efforts have briefly been made to give an update on the biological and therapeutic effects associated with this plant, and the main investigation regards the complete identification of the phytochemical contained in *M. officinalis*. More importantly, we present up-to-date research on the strategies and processes targeted toward the extraction and purification of its compounds for specific applications and purposes. Finally, as a perspective in the field, we also declare missing research gaps for future research groups interested in successfully extracting the phytochemicals from this plant. Herein, we also provide potential new strategies, emerging separation technologies and green solvents for the sustainable extraction of its components. 

## 2. An overview on the Phytochemicals Contained in *Melissa officinalis* L. and Their Related Biological Activities

*M. officinalis* is identified as a plant with remarkable pharmacological effects. To date, several studies have documented different pharmacological and biological effects of the extracts from this plant. Herein, we provide an updated scheme documenting all the pharmacological effects of *M. officinalis*. Very recently, Petrisor et al. [10] comprehensively reviewed the pharmacological effects of this herb, finding out that most of its biological activities are intrinsically related to its phenolic compounds [16]. Certainly, its phenolic compounds display exceptional antioxidant activity; however, antiproliferative [17], antiangiogenic [18], antimicrobial (toward fungi, bacteria and virus) [19,20], antianxiety [21], antidepressant [22], anti-Alzheimer’s [23], neuroprotective [24], and cardioprotective activities [25] are also among its discovered biological properties. 

The plant’s pharmacological properties have been associated with specific compounds, as specified in Table 2. For instance, betulinic acid and chlorogenic acid have been credited with the anticancer and antidiabetic properties, respectively. While more than one component has been related to other biological effects, e.g., antimicrobial properties (including antibacterial and antifungal) are a result of synergistic effects from different phytochemicals, such as geranial, neral, citronellal, β-caryophyllene, α-cadinol, geranyl acetate, ursolic acid, citronellal and geranyl acetate [26,27,28,29,30,31,32], as summarized in Table 2.

*M. officinalis* presents a great variety of phytochemicals belonging to major chemical classifications of phenolic acids, terpenoids and flavonoids [38]. Moreover, volatile compounds (such as geranial, neral, geraniol and citronellal), triterpenes (ursolic and oleanolic acid), phenolic compounds (such as rosmarinic, caffeic and protocatechuic acid) and flavonoids (such as rhamnocitrin, quercetin and luteolin) have been identified. To some extent, most bioactive phytochemicals have been profiled in the essential oil of *M. officinalis.* Table 3 enlists most of these compounds contained in the essential oil from the dried leaves. In general, major compounds (such as (E)-Caryophyllene, citronellal and geranial) can be present at a range of concentrations between 0.1 and 35%, while minor compounds (such as (2E)-Nonen-1-al, (E)-Nerolidol and (E)-α-Bergamotene) have been quantified to range from 0.1 to 3.6%. 

Triterpenes, which present three terpene units, are defined as non-volatile compounds. These compounds, which can own distinct sulfate groups linked to sugars or glucones, are the largest family of phytochemicals contained in natural plant-based products, and they are indeed present in *M. officinalis*. Ursolic, oleanolic and betulinic acids are found in large quantities in this herb at maximum concentrations of 11,234, 6151 and 170 μg/g [18], respectively, and while the rest of compounds have been identified, they have not been quantified accordingly. To some extent, the presence of such compounds may vary from one part to another in the plant, as shown in Table 4. In addition to the compounds reported in Table 3, other non-volatile components, such as disulfated ursene triterpenes and ursenic glycoside, have been documented by Mencherini et al. [39,40], who extracted them from dried stems and leaves. The same authors also reported the successful identification of three ursene triterpenes glycoside named as Melissioside A, B and C [39]. More recently, three different ursene triterpene glycosides (denominated as 23-sulfate triterpenoid glycoside ester of nigaichigosides) were discovered by Abdel-Naime et al. [41].

As for phenolic compounds, different phenolic acids (including caffeic, caftaric, chlorogenic, ferulic, gentisic, p-coumaric and rosmarinic acids) and flavonoids (apigenin, cymaroside, daidzein, hyperoside, isoquercetine, kaempherol, luteolin, myricetin, quercetin, quercetrol and rutin) have been identified in the leaves and aerial parts [42,43]. 

Over the course of this section, we have documented many phytochemical compounds with distinct bioactivity. However, less attention has been devoted to the extraction methods, which becomes relevant in terms of bioactivity degree and extraction efficiency. The following section reviews the main findings of extraction methods reported in the literature regarding the extraction of these phytochemicals. 

## 3. Recent Research on the Extraction and Purification of Phytochemicals from *M. officinalis*

To date, conventional solvent extraction has been the main pathway for extracting diverse phytochemicals from *M. officinalis*, as summarized in Table 5. For instance, Encalada et al. [17] successfully produced ethanolic and aqueous extracts containing mainly rosmarinic acid, which were subsequently assayed for cytotoxicity activity. By comparing both solvents (water and ethanol), it was noted that ethanol exhibited better extraction efficiency toward phenolic compounds and flavonoids, showing concentrations of about 3400 mg/100 g and 927 mg/100 g, respectively. Such values were much higher than the ones provided by aqueous extracts. These findings agree with previous reports supporting the exceptional affinity of polyphenols for ethanol [44]. Given the stronger polarity of water compared with ethanol, it seems that ethanolic solutions are suitable for extracting specific compounds with less polarity. According to Sun et al. [44], ethanol and ethanolic solutions are favorable for extracting some bioactive phytochemicals with a broad range of polarity, but not the most polar ones; in these latter compounds, water still stands as the most suitable solution. Certainly, both the nature and polarity of the solvents become relevant in extraction methods, especially in polyphenol extraction. A polar solvent displays better extraction efficiency thanks to the interactions (hydrogen bonds) between the polar sites of the bioactive compounds [45]. 

Compared with Encalada et al. [17], Magalhães et al. [46] reported higher concentration of rosmarinic acid (up to 5 mg/mL) in ethanolic extracts, in which a higher ethanol concentration was used for the extraction. Therefore, both studies confirm that ethanol seems to be the most favorable polar solvent for the targeted separation of phenolic acid. However, it is important to mention that some other components can also be extracted, e.g., during the extraction of phenolic compounds via ethanolic extraction, triterpenes have also been identified in the resultant extracts [18].

**Table 5:** Specific extraction of phytochemicals from *M. officinalis* using solvent extraction methods.

Compound	Solvents Used	Extraction Conditions	Remarks regarding the Study	Ref.
Rosmarinic acid	EtOH solutions * (50%) Aqueous solutions	Room temperature	Remarkable cytotoxicity activity of rosmarinic acid (1000 μg/mL) extract	[17]
Total phenolics	EtOH solutions (70%)	Room temperature, sonication (30 min)	Remarkable cytotoxicity activity and exceptional antioxidant properties	[47]
Rosmarinic acid (caffeic acid dimer)	EtOH solutions (80%)	25 °C, stirring (150 rpm)	Notable tumor inhibition activity of phenolic extract (5 mg/mL)	[46]
Total phenolics	EtOH and methanolic solutions	-	Acceptable antioxidant activity and good activity towards lipid peroxidation	[48]
Citronellal, thymol, citral, β-caryophyllene	Aqueous extracts	100 °C	Notable antioxidant properties	[49]
Rosmarinic acid, caftaric acid, gentisic acid, chlorogenic acid, caffeic acid, p-coumaric acid, ferulic acid, sinapic acid, hyperoside, isoquercitrin, rutin, myricetin, fisetin, quercitrin, quercetol, luteolin, kaempferol, apigenin	EtOH solutions (70%)	Room temperature	The resultant phenolic-enriched extract displayed potential chemo-preventive activity	[18]
Cinnamic acid	EtOH solutions (96%)	Room temperature	The extract exhibited cardioprotective effects due to antioxidant properties	[25]
Rosmarinic acid	EtOH solutions (70%)	Room temperature	Resultant extract exhibited anxiolytic and antidepressant activity	[21]
Rosmarinic acid, triterpenoids, ursolic acid, oleanolic acid	Ethyl acetate, methanol, hexane, water	Room temperature, 24–48 h	Methanolic extract displayed the best anxiolytic activity	[24]
Total phenolics and flavonoids	EtOH solutions (99.9%)	Room temperature, 72 h	Remarkable anti-leishmania and anti-trypanosoma activities were observed in the resultant extract	[50]
Phenolic compounds	EtOH solutions (75%)	Room temperature, 48 h	The resultant compounds revealed analgesic effect in rat models	[51]
Phenolic compounds	EtOH solutions	-	Anti-insomnia effect was observed in the enriched phenolic extracts	[52]
Phenolic compounds	EtOH solutions	Room temperature	The ethanolic extract displayed a reduction effect on glucose levels in rats	[53]
Rosmarinic acid and salvianolic acids	Methanolic solutions (70%)	37 °C	The resultant extracts exhibited visible GSK-3β-inhibitory activity	[23]
Rosmarinic acid	EtOH solutions (70%)	-	The resultant extract exhibited symptomatic benefits in the gastrointestinal tract	[54]
Caffeic acid, p-coumaric acid, rosmarinic acid	Aqueous extracts	100 °C	An antiviral effect was observed in the extract.	[55]
Phenolic compounds, alkaloids	EtOH solutions (70%)	-	Positive antifungal activities were observed in the obtained extract	[56]

* EtOH: ethanol.

In a practical study, Awad et al. [57] used four different solvents (ethyl acetate, methanol, hexane and water) to study the effects of their polarity on the extraction of specific phytochemicals. To some extent, methanol was found to be the most suitable solvent for the simultaneous extraction and isolation of rosmarinic acid, triterpenoids, ursolic acid and oleanolic acid, in which most of the extracts contained rosmarinic acid as the major active element. Importantly, methanolic extract enriched in rosmarinic acid also acted as in vitro inhibitor of rat brain GABA transaminase (40% inhibition at 100 μg/mL), which is generally related to specific illnesses such as anxiety, epilepsy and other neurological disorders. In the supporting Award [57] outcomes, Gürbüz et al. [23] also reported the presence of rosmarinic acid and salvianolic acids in methanolic extracts, which also confirmed a potential effect of GSK-3β-inhibitory activity related to Alzheimer’s disease.

Regarding the extraction of volatile compounds from essential oils, Ehsani et al. [49] extracted citronellal (37.33%), thymol (11.96%), citral (10.10%) and β-caryophyllene (7.27%) via hydro-distillation. The authors demonstrated that the physicochemical composition of *M. officinalis* essential oils is composed of around 85% volatile components, and thus, they provide exceptional antioxidant properties and antibacterial properties. In a different study, Chung et al. [58] reported the presence of large amounts of monoterpene, sesquiterpene and some other carbonyl-based phytochemicals in essential oils. The authors reported the successful production of such essential oils via steam distillation, followed by extraction with distilled water and diethyl ether for 2 h at atmospheric pressure. 

More recently, El Ouadi et al. [19] experimented with the production of essential oil from *M. officinalis* using hydro-distillation (average yield of 1%). Interestingly, the authors detected P-mentha-1,2,3-triol as the main volatile compound (by 13.1%) in the resultant essential oil, which was later tested for its antifungal activities against *Bcinera*, *Pexpansum* and *Rstolonifer*, along with a bio-antifungal preservative for post-harvest diseases of fruits (e.g., apples). The authors also reported the presence of other relevant phytochemicals, including P-menth-3-en-8-ol (8.8%), pulegone (8.8%), piperitynone oxide (8.4%) and 2-piperitone oxide (7.3%).

## 4. Perspectives and Research Gaps: Potential of New Strategies, Emerging Separation Technologies and Green Solvents for Sustainable Extraction

In the last section of this review, we confirmed the presence of a huge number of phytochemicals (Table 3 and Table 4) with different biological and pharmacological effects in *Melissa officinalis* L. (see Table 2). To some extent, the efforts of the research community have led to evidence that solvent extraction is the most common strategy for producing enriched extracts, which have been subsequently tested for further bioactivity evaluation. Herein, polar solvents (such as ethanol and methanol) are reported as the most suitable for the extraction of specific phenolic compounds (e.g., rosmarinic acid). However, this review identifies that the research community has been focused on the evaluation of the pharmacological effects of alcoholic extracts without the optimization of process variables, and with no further fractionation and purification of the obtained extracts. Regarding the latter point, scientists could implement the following new strategies, emerging separation/extraction techniques and green solvents for the sustainable extraction of such phytochemicals:

- *Solvent extraction*: If the research community still applies solvent extraction as the primary method of recovering extracted compounds from this plant, calculating the partition coefficient (logP) of the solvents is a must since the solute is distributed between two immiscible solvents. Furthermore, determining the solubility (logS) of target compounds in the solvents is also suggested to obtain high recovery yields.
- *Membrane separation techniques*: These processes, such as ultra- and nanofiltration, use a perm-selective barrier based on the molecular sieving mechanism for the separation of compounds [59,60]. These latter physical separation technologies have been used for the fractionation and purification of various biological active compounds, such as phenolic compounds, low-molecular-weight carbohydrates, proteins, flavonoids, glycosides and anthocyanins, among others, from several sources, including natural extracts, agro-food by-products and wastes, and fermentation systems [61,62,63,64,65]. *Thus far, there are no reports documenting the application of such technologies in the purification of phytochemicals of M. officinalis*. Eventually, considering the molecular weight of rosmarinic acid (ca. 360 g/mol), a nanofiltration membrane with a molecular weight cut-off ranging from 150 to 300 kDa would be enough to concentrate this compound once contained in aqueous and alcoholic extracts [66]. Herein, preliminary filtration steps based on microfiltration and ultrafiltration would be needed to remove other undesired molecules from the raw extract, as reported in the literature [67,68,69].
- *Emerging extraction techniques*: To date, distinct emerging extraction techniques have been developed, such as microwave, ultrasound, pulsed electric-assisted extraction, supercritical/subcritical fluids and pressurized liquids, among others, which have emerged as advanced pathways for extracting different types of biomolecules from plant-based sources [6,13,70]. The application of such processes enables the handling of different operating conditions, such as solvent-to-solid ratio, irradiation time, pH, temperature, agitation speed, microwave power, pressure and ultrasound intensity, for optimization of the overall extraction process. *Thus far, there are no reports documenting experimentation using any of these techniques for the extraction of phytochemicals from this plant*. Importantly, before applying any of aforementioned techniques, the application of any pre-treatment of the plant source, such as enzyme-assisted extraction [71] or hydrodynamic cavitation (HC) [72,73], could be beneficial to obtain higher extraction yields, e.g., enzyme treatment is used to break lignocellulosic matter, making more phytochemicals available for extraction. While HC based on the cavitation phenomenon boosts extraction efficiency due to the increased mass transfer rate between the substrate and solvent, while the disintegration of solids/lowering of particle size occurs following cell wall rupture thanks to the intense implosion of cavitation bubbles. On the other hand, special attention should be paid to aspects of uncontrolled oxidation reactions that can take place during cavitation-assisted processes that cause qualitative changes in as-obtained extracts [74].
- *Ionic liquids*: Given the content of volatile and nonvolatile compounds, in addition to the phenolic compounds identified in *M. officinalis, a* selective solid/fluid extraction method could be designed using a neoteric solvent such as supercritical CO_2_ (SCO_2_) or ionic liquids, in order to separate triterpenoids, essential oils and target acids from leaves and stems. Ionic liquids are recognized for their solvent power, polarity and hydrophobic/hydrophilic behavior using hydrophilic-based imidazolium ionic liquids. For instance, Claudio and coworkers [75] improved their extraction yields of oleanolic acid extracted from olive tree leaves by up to 2.5 wt%. Yang and coworkers [76] used the same group of ionic liquids to extract chlorogenic acid from ramie (*Boehmeria nivea* L.) leaves, with a maximum extraction efficiency of 96.18%. Rosmarinic acid, which is also present in *M. officinalis*, has been successfully extracted from *Rosmarinus officinalis* [77] from perilla seeds using hydrophilic ionic liquid due to the interaction with the cellulose of the cell wall [78]. Therefore, similar hydrophilic ionic liquids should be explored to extract such bioactive compounds from *M. officinalis*.

The solid/supercritical fluid extraction of caffeine from coffee beans has been reported [79], which could potentially be applied to *M. officinalis* leaves or steams; however, a purification process using SCO_2_ after the solid/liquid extraction process has been reported for organic compounds [80] in liquid/dense gas extraction, or using a membrane contactor to avoid the mass transfer drawback of the liquid/gas extraction [81]. Using EtOH:water (50/50 v/v) rosemary extract, Lefebvre and coworkers [82] obtained carnosic acid and rosmarinic acid using SCO_2_, and Chadni and coworkers obtained 8 mg/g of rosmarinic acid using SCO_2_ from the organic phase after the distillation process of *Salvia sclarea*. 

A purification step for organic compounds from water or water/EtOH extract has also been studied using hydrophobic ionic liquids [83], and this purification step could take place after the processes shown in Table 5, which are used to find a purer extract that leaves behind a phenolic compound. Yan-Ying and coworkers [84] used [PF_6_]-based hydrophobic ionic liquids to obtain ferulic acid and caffeic acid from aqueous solution; however, the use of ionic liquid to separate or purify phytochemicals from *M. officinalis* is still a field that is not covered in the literature.

- *Deep eutectic solvents*: As chemistry evolves, new extraction techniques and solvents are developed that provide eco-friendly alternatives to conventional extraction procedures. For instance, most of the conventional solvents (methanol, hexane, cyclohexane, etc.) tend to display related toxicity to human beings and the environment. Very recently, new green solvents, such as deep eutectic solvents (DESs), have emerged as an eco-friendly alternative for targeted extractions. DESs are a combination of two or three inexpensive and safe chemicals (e.g., choline chloride, urea glucose, proline, xylitol among many others), which can be self-assembled by hydrogen bonds [85,86]. To date, antioxidants [87], phenolic compounds [88], capsaicins [89], terpenoids [90], heavy metals (Ni, Zn, Pb) [91,92,93] and pharmaceuticals [94], among many other components, have been successfully extracted via DESs from different source systems. *Thus far, there are no reports documenting the experimentation of any eutectic solvent for the extraction of phytochemicals from M. officinalis*. Researchers need to carefully select the type of DES system based on its nature (hydrophilic or hydrophobic—hDESs) [95] and the polarity of the target phytochemical. Uncommon selectivity, compared to organic solvents, can be obtained through the “tuning” of extracts’ properties using DESs tailored to specific applications [96]. The latest development in this field relates to the *ifcastron-situ* formation of DESs, assisted by mechanical mixing (a mechano-chemical approach) [97]. In this process, only one of the pre-defined DES components in solid state is mixed with powdered plant material. The DES is formed with the target antioxidant (rosmarinic acid) that is primarily present in the plant material.

## 5. Conclusions and Research Gaps

Over the course of this review, we complied the most recent literature dealing with the presence of phytochemicals in *M. officinalis* and their related biological and pharmacological effects, as the usage of this plant has been promoted for many years as part of traditional medicine. Additionally, this review analyzed one of the most important aspects of the extraction of phytochemicals from the plant, revealing that ethanol has been the preferred polar solvent in conventional solvent extraction. To some extent, the usage of ethanol as a polar solvent results in the successful extraction of rosmarinic acid since it displays a high affinity for such solvents, according to several studies [17,18,21,46,55]. 

By reviewing the extraction procedures used in all the studies, it was observed that most of the experimental works are mainly focused on biological and pharmacological studies, while minimal emphasis is devoted to the analysis of the extraction process, e.g., authors rarely report the real concentration of the compounds or target analytes. Additionally, most of the studies lack data on extraction yield or efficiency. In addition to this, there are no studies about the proper fractionation of the resultant alcoholic extracts. Therefore, there is still a need to identify the main compounds associated with precise bioactivity.

Finally, most of the authors do not report the pre-conditioning of *M. officinalis* samples before extraction. Here, major attention is needed, since drying and milling affect the final particle size of the dried samples, with a strong effect on the extraction yield and the resulting concentration of the phytochemicals in the extract. As for the extraction process, there is a need to optimize the extraction conditions.

Further studies should also focus on emerging extraction and separation techniques, such as the ones based on the cavitation phenomenon or membrane-assisted processes, and the replacement of organic solvents with “green” alternatives—for example, DESs. On the other hand, extracts obtained from a liquid phase are not the final desired product. Thus, well-established processes should include aspects of solvent recovery, as well as resource and energy cost optimization.

## Figures and Tables

**Table 1:** Ethno-pharmacological applications of *M. officinalis* in different countries. Adapted from [11].

Country	Part of the Plant	Dosage Form	Medicinal Uses/Illness Treated
**Austria**	Leaves	Tea, essential oil (external application)	Curing gastrointestinal, nervous, hepatic and biliary ailments
**Bolivia**	Aerial part	Infusion	Curing heart ailments
**Bosnia and Herzegovina**	Leaves	Oral preparations	Curing insomnia, restlessness, arrhythmia, increased lactation, flatulence, depressions, morning sickness, diarrhea, migraine and rheumatism; strengthening the body; internal purification; blood purification
**Brazil**	Leaves	Infusion tea	Sedative for children; curing stomach disturbances, bad cold, cough, infection and fever
	Leaves	Bath	Wound healing (external use)
	Roots	Decoction	Curing bad cold and cough
**Bulgaria**	Leaves	Infusion	Sedative, hypotensive and spasmolytic
**Croatia**	Leaves	Infusion	Curing sore throat and cough
**Denmark**	Aerial parts	Infusion	Curing sleeplessness caused by heart break, melancholy and sadness
**Ecuador**	Stems, leaves	Infusion	Relaxant, curing insomnia
**Greece**	Aerial parts	Infusion, decoction	Blood circulation and heart stimulant; curing hypertension, earaches, bloating, dyspepsia, spasm, headache, depression, dizziness, migraine and common cold; decreasing cholesterol and uric acid; brain stimulant; calmative
**India**	Leaves	Infusion, decoction	Promoting memory;
**Iran**	Leaves	Infusion (oral)	curing depression, anxiety, psychosis, palpitation, obsession, dementia, epilepsy, stroke, tremor, paralysis, migraine and vertigo, syncope asthma, diabetes fevers, hiccups, joint inflammation and pain, cancer, halitosis, aphtha, rabies, gastrointestinal problems and piles; menorrhagia exhilarant; cardiac and gastric tonic; mild sedative; memory enhancer; antidote; disinfectant
	Leaves	Oil (inhalation)	Treatment of nightmares
	Leaves	Infusion (external use)	Curing conjunctivitis and lack of eyesight
**Iraq**	Leaves	Tea	Diuretic; analgesic; treats headache and toothache; galactogenic
**Italy**	Leaves	Poultice infusion	Wound healing Curing abdominal pains
	Leaves	Compress of crushed fresh leaves	Healing insect bites
**Jordan**	Aerial parts	Infusion	Sedative; carminative; antispasmodic; curing abdominal pain, digestive and gynecological disorders and arthritis
**Kosovo**	Aerial parts	Infusion	Curing abdominal pains during pregnancy;
**Lebanon**	Aerial parts	Decoction	neuro-relaxant; strengthening the heart; curing migraine and stomach disorders; enhancing memory
**Morocco**	Aerial parts	Infusion	Spasmolytic, depurative and tranquilizing; heart tonic; cholagogue
**Palestine**	Aerial parts	Infusion	Antimicrobial
**Patagonia**	Aerial parts	Infusion	Sedative
**Peru**	Leaves	Infusion	Sedative and hypotensive
**Poland**	Leaves	Infusion	Nervous excitability; curing vegetative neurosis, tension, anxiety, motor agitation and menopause
**Portugal**	Aerial part, stems and flowers (fresh or dried)	Infusion	Relaxation (insomnia, nervousness and spasms)
**Republic of Macedonia**	Leaves	Tea, oil	Curing heart problems and headache
**Spain**	Leaves	Tea, oil	Exhilarant; antidote; emmenagogue pain killer; curing intestinal ulcers, gripes, heart palpitations caused by consumption of toxic mushrooms, and orthopnea
**Turkey**	Aerial part	Infusion Decoction	Curing cancer, asthma, cardiovascular diseases, nephritis, forgetfulness, diabetes, cold, bronchitis and enteritis; antiseptic; antispasmodic; memory-enhancing

**Table 2:** Biological activities associated with specific phytochemicals contained in *M. officinalis*.

Activity	Compounds Related to Such Activity:	References
Antibacterial and antifungal	Geranial, neral, citronellal, β-caryophyllene, α-cadinol, geranyl acetate, ursolic acid	[26,27,28,29,30,31,32]
Insecticidal	Citronellal, geranyl acetate	[29,31,32,33]
Anti-inflammatory	β-caryophyllene, betulinic acid, caffeic acid, rosmarinic acid, chlorogenic acid, p-coumaric acid, cymaroside, rutin	[29,34,35]
Antioxidative	β-caryophyllene, ursolic acid, caffeic acid, caftaric acid, rosmarinic acid, ferulic acid, chlorogenic acid, p-coumaric acid, cymaroside, rutin	[29,33,35]
Hepatoprotective	α-cadinol, oleanolic acid	[30,36]
Antiviral	Betulinic acid, oleanolic acid	[34,36]
Anticancer	Betulinic acid	[34]
Antidiabetic	Chlorogenic acid	[37]

**Table 3:** Major and minor volatile bioactive compounds identified in essential oil of *M. officinalis*. Adapted from [10].

Major Compound	Chemical Structure	Minor Compounds ^1^	Chemical Structure
(E)-Caryophyllene	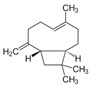	(2E)-Nonen-1-al	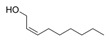
Caryophyllene oxide	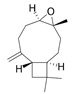	(E)-Nerolidol	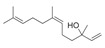
Citronellal	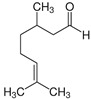	(E)-α-Bergamotene	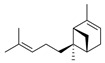
Geranial (citral A)	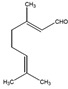	(E)-β-Ionone	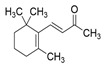
Geranyl acetate	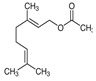	(E)-β-Ocimene	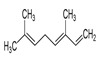
Neral (citral B)	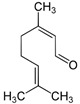	(E-E)-Geranyl linalool	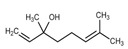
α-Cadinol	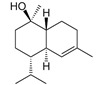	(Z)-β-Ocimene	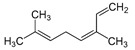
α-Copaene	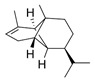	1,2-Benzenedicarboxilic acid, butyl 2-methylopropyl ester	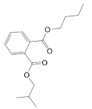
β-Caryophyllene	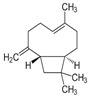	1,8-Dehydro-cineol	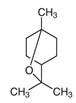
		14-Hydroxy-9-epi-(E)-caryophyllene	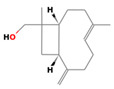
		1-Octen-3-ol	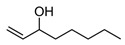

^1^ Full list of minor compounds in the essential oil can be found in [10].

**Table 4:** Triterpenes identified in *M. officinalis*. Adapted from [10].

Triterpene	Part of Plant
Ursolic acid *	Aerial part
Oleanolic acid **	Aerial part
Betulinic acid ***	Aerial part
3β,16β,23-Trihydroxy-13,28-epoxyurs-11-ene-3-O-β-D-glucopyranoside	Leaves and stems
3,23-Disulfate ester of 2α,3β,19α,23-tetrahydroxyurs-12-en-28-oicacid	Leaves and stems
3,23-Disulfate ester of 2α,3β,19α,23-tetrahydroxyurs-12-en-28-oicacid 28-O-β-D-glucopyranoside	Leaves and stems
3,23-Disulfate ester of2α,3β,23,29-tetrahydroxyolean-12-en-28-oicacid	Leaves and stems
3,23-disulfate ester of 3β-23,29-trihydroxyolean-12-en-28-oic acid	Leaves and stems
3,23-Disulfate ester of 2α,3β-23,29-tetrahydroxyolean-12-ene-28-oicacid	Leaves and stems
23-sulfate ester of 2α,3β,19 α,23-tetrahydroxyurs-12-en-28-oic acid	Leaves and stems
23-sulfate ester of 2α,3β,19 α,23-tetrahydroxyurs-12-en-28-oic acid 28-O-β- D-glucopyranoside	Leaves and stems
Melissioside A, B and C	Leaves and stems

* 5577–11,234 µg/g, ** 915–6151 µg/g, *** 12–170 µg/g. Aerial parts of plants refers to the plant’s structures above ground, such as stems, leaves, petioles, flowers, fruits and seeds. Stem refers to the ascending portion of the axis, bearing branches, leaves, flowers and fruits. Leaves are a lateral, flattened structure that grows from the stem.

## Data Availability

The data are contained within the article.
